# A note on arterial to venous oxygen saturation as reference for NIRS-determined frontal lobe oxygen saturation in healthy humans

**DOI:** 10.3389/fphys.2013.00403

**Published:** 2014-01-22

**Authors:** Henrik Sørensen, Niels H. Secher, Peter Rasmussen

**Affiliations:** Department of Anesthesia, The Copenhagen Muscle Research Center, Rigshospitalet, University of CopenhagenCopenhagen, Denmark

**Keywords:** cerebral oxygenation, humans, hypocapnia, hypercapnia, hypoxia, jugular vein, NIRS

Near infrared spectroscopy (NIRS) offers non-invasive assessment of oxygenation within the human brain (S_c_O_2_) by appreciating the different absorption of near infrared light by hemoglobin and oxyhemoglobin (Jobsis, 1977). Since the length the light has passed when traveling from the skin to the cortex and then returning to the skin remains unknown, there is a need to adjust the signal according to an assumed ratio between the arterial vs. venous blood that is appreciated. Apparently, most NIRS devices use a fixed reference ratio between the arterial (25%) and venous contribution (75%) to the signal despite not eligible mismatch (Bickler et al., 2013). This assumption is based on anatomical evidence (Pollard et al., 1996), but might be confounded by changes in cerebral blood volume during, e.g., hypoxia and changes in the arterial carbon dioxide tension (P_a_CO_2_) (Ito et al., 2005). A standard reference arterial to venous ratio may therefore not exist for application of NIRS to determine S_c_O_2_ in humans. Thus, an estimate of cerebral capillary hemoglobin oxygen saturation (S_cap_O_2_) to express cerebral oxygenation is based on 50% jugular and arterial saturations (Gjedde et al., 2005) and has been reported to follow changes in S_c_O_2_ (Rasmussen et al., 2007). In this report we made a meta-analysis on published data (Rasmussen et al., 2007; Sørensen et al., 2012, in press) in order to evaluate which ratio between arterial and internal jugular venous hemoglobin saturation that fits best to the concomitant determined S_c_O_2_ in healthy humans exposed to a wide range of interventions.

Thirty seven subjects [age 27(9) years, height 181(10) cm, mass 79(13) kg, mean (*SD*)] were catheterized in the right internal jugular vein with the tip of the catheter advanced to the bulb of the vein and with a catheter in the brachial artery of the non-dominant arm, while S_c_O_2_ was monitored by the Invos Cerebral Oximetry (Covidien, Mansfield, MI). Arterial partial pressure for oxygen and carbon dioxide, and oxygen saturations were measured in the jugular and arterial blood (P_a_O_2_; P_a_CO_2_; S_a_O_2_; S_j_O_2_) (ABL-800, Radiometer, Brønshøj, Denmark). The subjects were exposed to hypoxia (F_i_O_2_ = 10%; *n* = 23), inspiration of 100% oxygen (*n* = 8), atmospheric air (*n* = 37), hypercapnia (F_i_CO_2_ = 5%; *n* = 8) and asked to hyperventilate (~2–3 kPa reduction in P_a_CO_2_; *n* = 32) with separate controls. By linear regression the contribution from arterial and jugular blood to S_c_O_2_ was estimated and R-squared (*R*^2^) and root mean square error (RMSE) between S_c_O_2_ and the arterial fraction in the reference saturation were calculated (SAS Institute Inc., Cary, NC). Only data points where S_j_O_2_ ≤ S_c_O_2_ ≤ S_a_O_2_ were included. All reference saturations were calculated, e.g., S_cap_O_2_ = 0.50 · S_a_O_2_ + 0.50 · S_j_O_2_. The following equation, 0 = S_c_O_2_ – [a · S_a_O_2_ + (a − 1) · S_j_O_2_], was used to calculate the arterial fraction (a) for which the difference between S_c_O_2_ and the reference saturation was zero.

S_a_O_2_ and S_j_O_2_ ranged from 70 to 100% and 33 to 87%, respectively, while S_c_O_2_ ranged from 48 to 95%. P_a_CO_2_ was manipulated from 1.6 to 6.3 kPa. According to the linear regression analysis, S_c_O_2_ demonstrated a correlation to a wide range of ratios between the arterial and venous hemoglobin saturations. The highest RMSE was obtained when it was considered that there was no arterial contribution to S_c_O_2_ and the RMSE became gradually lower when the arterial contribution was considered to increase (Figure 1A). The lowest RMSE was observed for a 75% arterial and 25% jugular venous blood contribution to S_c_O_2_(RMSE = 2.70; *R*^2^ = 0.644; *P* < 0.0001; Figure 1A). For the often-used calibration ratio (25% arterial) *R*^2^ was 0.505 (*P* < 0.0001) with a RMSE of 4.233. In contrast, S_cap_O_2_ (50% arterial) had a *R*^2^ of 0.606 (*P* < 0.0001; RMSE = 3.276; Figure 1A). Zero was within the 95% confidence interval only with a calculated 40–50% arterial contribution to the reference ratio. When S_c_O_2_ was compared with the calibration ratio, the mean difference was zero in 2.9% of the blood samples, whereas it was 10.5% when S_cap_O_2_ was used as reference (Figure 1B).

**Figure 1:**
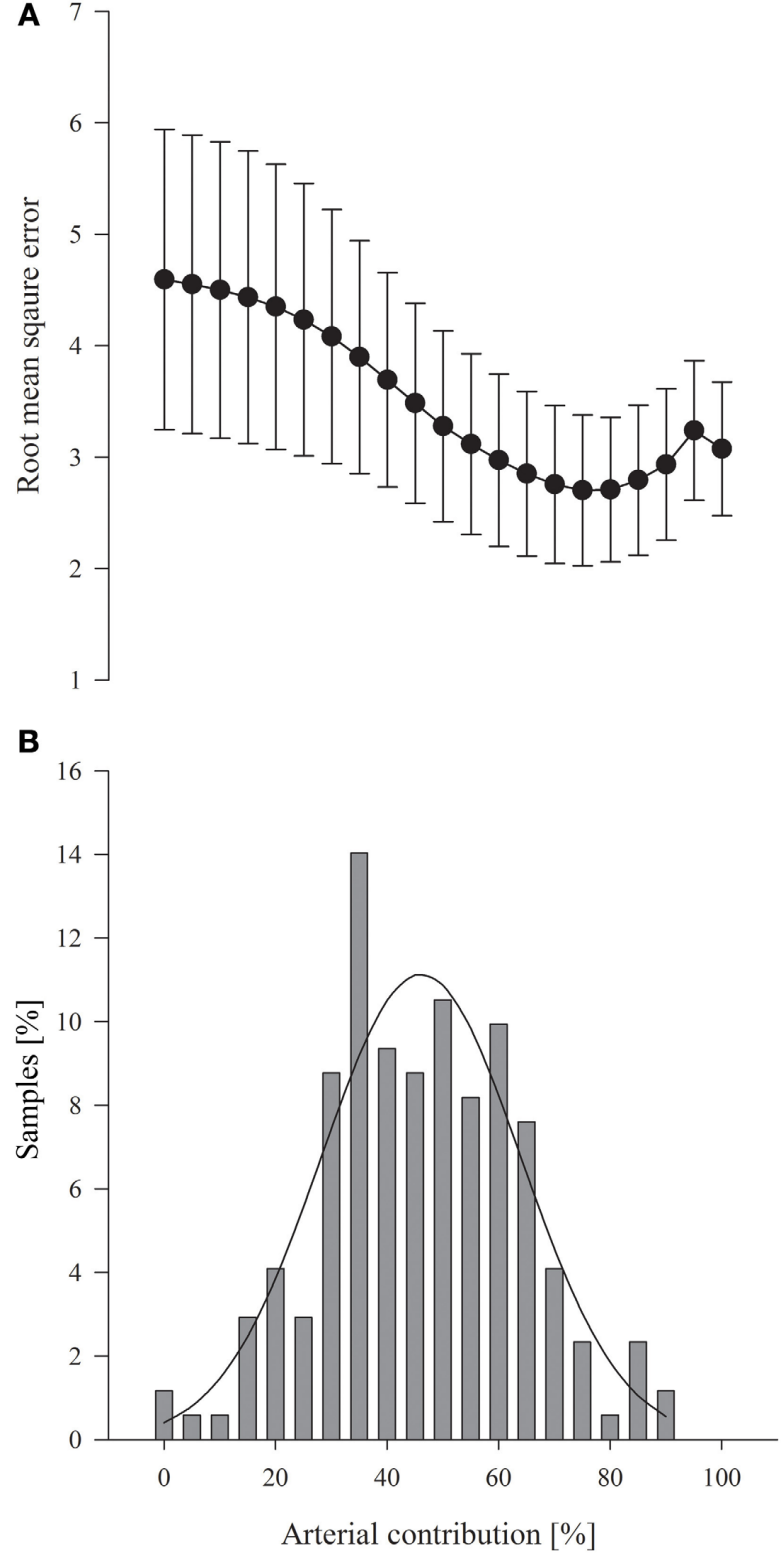
**(A)** Root mean square error for the arterial fraction determined by the linear regression analysis. Values are mean (95% confidence interval). **(B)** Zero difference between the Invos-determined cerebral oxygenation (S_c_O_2_) and the calculated reference saturation related to the considered arterial contribution for 171 blood samples; average arterial contribution 46 ± 17% (*SD*). Solid line represents the mean distribution

This meta-analysis of published data suggests that the optimal reference ratio has a larger arterial contribution than the ratio defined by anatomical models and likely incorporated in most NIRS devices (Pollard et al., 1996; Bickler et al., 2013). The subjects were not only exposed to conditions that changes the venous oxygen saturation, i.e., hyperventilation, but also to conditions known to affect arterial and venous cerebral blood volume and oxygen content without influencing extra-cerebral blood (Ito et al., 2005). From these interventions, linear regression analysis demonstrated that the correlation between S_c_O_2_ and the arterial contribution to the reference saturation was highest when a 75% arterial contribution was considered (*R*^2^ = 0.644) and also for that ratio the RMSE was the lowest (2.70) (Figure 1A). The calibration ratio (25% arterial) typically incorporated in the algorithms had a *R*^2^ of 0.505 and a RMSE of 4.233 while S_cap_O_2_ demonstrated a stronger correlation with a *R*^2^ of 0.606 (*P* < 0.0001; RMSE = 3.276). In addition, the mismatch between S_c_O_2_ and the reference ratio was more likely to be zero when a more arterial weighted reference was applied (Figure 1B). Thus, our findings demonstrate that S_cap_O_2_ is an accurate reference for S_c_O_2_ at least when determined with the Invos apparatus.

Similar to these findings, no eligible mismatch between S_c_O_2_ values and the calibration ratio (25% arterial) is observed in five different NIRS devices used to determine cerebral oxygenation in healthy subjects exposed to isocapnic hypoxemia (Bickler et al., 2013). Hypercapnia and isocapnic hypoxemia induce cerebral vasodilation and affect cerebral blood volume mainly by an increase in the arterial fraction (Ito et al., 2005). Thus, it is likely that the illuminated area of the brain encompasses more arterial blood and that could explain the aggravated mismatch between S_c_O_2_ and the calibration ratio during hypoxia (Bickler et al., 2013). Cerebral blood flow and jugular venous oxygen saturation are decreased with hyperventilation that reduce the calibration ratio more compared to S_cap_O_2_. Interestingly, when healthy humans hyperventilate the S_c_O_2_ overestimated the calibration ratio by 11.2% while the mean bias was only 0.2% for S_cap_O_2_ (Sørensen et al. unpublished). Thus, the mismatch between the calibration ratio and S_c_O_2_ is aggravated when only jugular venous oxygen saturation is altered, which indicates that S_c_O_2_ accounts for more than 25% arterial blood and that evaluations of reference saturations for NIRS must also involve conditions known to affect arterial oxygen content.

The arterial to venous balance within the brain may differ between individuals and explains the inter-individual variation in absolute S_c_O_2_ readings (Rasmussen et al., 2007; Bickler et al., 2013), especially the heterogeneity of blood vessels in the illuminated area of the brain seems to affect light absorption because the photons are “lost” in major blood vessel, e.g., the sagittal sinuses (Kishi et al., 2003). Other factors affecting light absorption and thereby the S_c_O_2_ readings may be variation in skull thickness and amount of cerebrospinal fluid (Yoshitani et al., 2007; Strangman et al., 2014). Also, skin pigmentation (Bickler et al., 2013) and degradation products of heme can affect S_c_O_2_ because of competitive absorption of light (Madsen et al., 2000).

Despite absolute S_c_O_2_ values are not comparable and exhibit large variations when compared to the calibration ratio, NIRS offers a unique non-invasive method for assessment of cerebral oxygen delivery vs. consumption and its clinical utility relies on changes from baseline rather than on absolute values (Murkin et al., 2007). Yet, at present the NIRS technology for evaluation of S_c_O_2_ is limited when skin blood flow is affected either by scalp ischemia or administration of sympathomimetic agents that affect skin blood flow (Davie and Grocott, 2012; Sørensen et al., 2012).

In summary, this report suggests that the reference saturations applied when using Invos cerebral oximetry should be weighted more to arterial hemoglobin saturation than accepted by anatomical models.

## Author contributions

All authors contributed equally to the design, data analysis, and interpretation, drafting the manuscript and critical revision. All authors approved the final version before submission.
